# Determination of AAV properties by single amino acids: Go(o)d is in the details

**DOI:** 10.1016/j.omtm.2022.09.006

**Published:** 2022-09-29

**Authors:** Olena Maiakovska, Conradin Baumgartl, Dirk Grimm

**Affiliations:** 1 Department of Infectious Diseases/Virology, Section Viral Vector Technologies, Medical Faculty, University of Heidelberg, Center for Integrative Infectious Diseases Research (CIID), BioQuant, 69120 Heidelberg, Germany; 2 German Center for Infection Research (DZIF), Partner Site Heidelberg, 69120 Heidelberg, Germany; 3 German Center for Cardiovascular Research (DZHK), Partner Site Heidelberg, 69120 Heidelberg, Germany

Adeno-associated virus (AAV) has gathered substantial attention as a gene transfer vector in humans owing to its numerous assets, including amenability to genetic engineering and repurposing. Equally attractive is the wealth of natural AAV variants that can be harnessed as vectors or molecularly evolved into designer AAVs. A particularly interesting group of 15 variants dubbed AAVHSC, related to AAV clade F and differing from each other by one to four amino acids, was isolated from CD34+ human hematopoietic stem cells. The latest work from Smith and colleagues now finds that a specific member of the AAVHSC group, AAVHSC16, is distinguished by its lack of binding to membrane-exposed galactose;^1^ the latter acts as a primary receptor for the other AAVHSCs and the clade F prototype AAV9. Mutagenesis and residue swapping identified two distinct amino acids at positions 501 and 706 as most critical for AAVHSC16’s unique phenotype. The finding that AAVHSC16 is broadly active but liver detargeted in peripherally injected non-human primates (NHPs), coupled with the absence of hepatotoxicity, implies significant potential for clinical translation. While this conclusion awaits *in vivo* validation with larger animal numbers, additional work is also warranted to dissect the biology of this remarkable AAV variant including receptor usage and immunoreactivity.

For decades, AAV has gradually taken center stage among all viruses that are engineered as vectors for human gene therapy, driven by the enormous variety of natural AAV isolates in different species. When engineered as vectors, these can differ in pivotal parameters, such as transduction specificity and/or efficiency, or reactivity with anti-AAV antibodies. This fostered efforts to concurrently (1) dissect the sequence-structure-function relationships that underlie AAV’s properties and (2) harness this knowledge for the molecular evolution or rational design of next-generation vectors. To this end, a wealth of strategies were pursued including, for instance, site-directed mutagenesis of the AAV2 capsid gene. Changing ∼8% of all amino acids enabled the Muzyczka lab to identify positions that impact capsid assembly, DNA packaging, or infectivity.^2^ Alternatively, Vandenberghe and colleagues performed a meta-analysis of AAV sequence data and mutagenized residues that varied in otherwise conserved positions (“singletons”). One AAV6 variant, AAV6.2, in which two singletons were reverted, performed exceptionally well following intravenous or intramuscular injection in mice and surpassed other clade members including AAV1.^3^ Similarly, our own group isolated and compared a set of capsid sequences derived from another parvovirus, human bocavirus 1 (HBoV1), from primary human material. Identification and targeted mutation of singletons revealed their role in transduction, capsid assembly, or DNA packaging of HBoV1 vectors.^4^

Another approach reported by several groups utilizes directed evolution to create complex capsid libraries comprising random point mutations introduced by error-prone PCR. Akin to the aforementioned work, this enabled them to identify residues critical for, e.g., heparin binding or antibody evasion.^5^ A powerful permutation of this strategy by the Nakai lab introduced DNA barcoding technology into the AAV field and showcased its potential through massively parallel screening of a double-alanine mutant AAV9 library.^6^ Interestingly, and relevant in the context of the new work by the Francone lab, this identified a role of 26 residues in binding of AAV9 to its primary receptor N-terminal galactose, including a surface-exposed tyrosine at position 706. Finally, we note recent reports by multiple groups including the Church and Kelsic labs that explored deep learning to achieve the two above-mentioned goals, i.e., a better understanding of single residues in AAV biology concurrent with rational design of next-generation vectors.^7^

**Figure 1:**
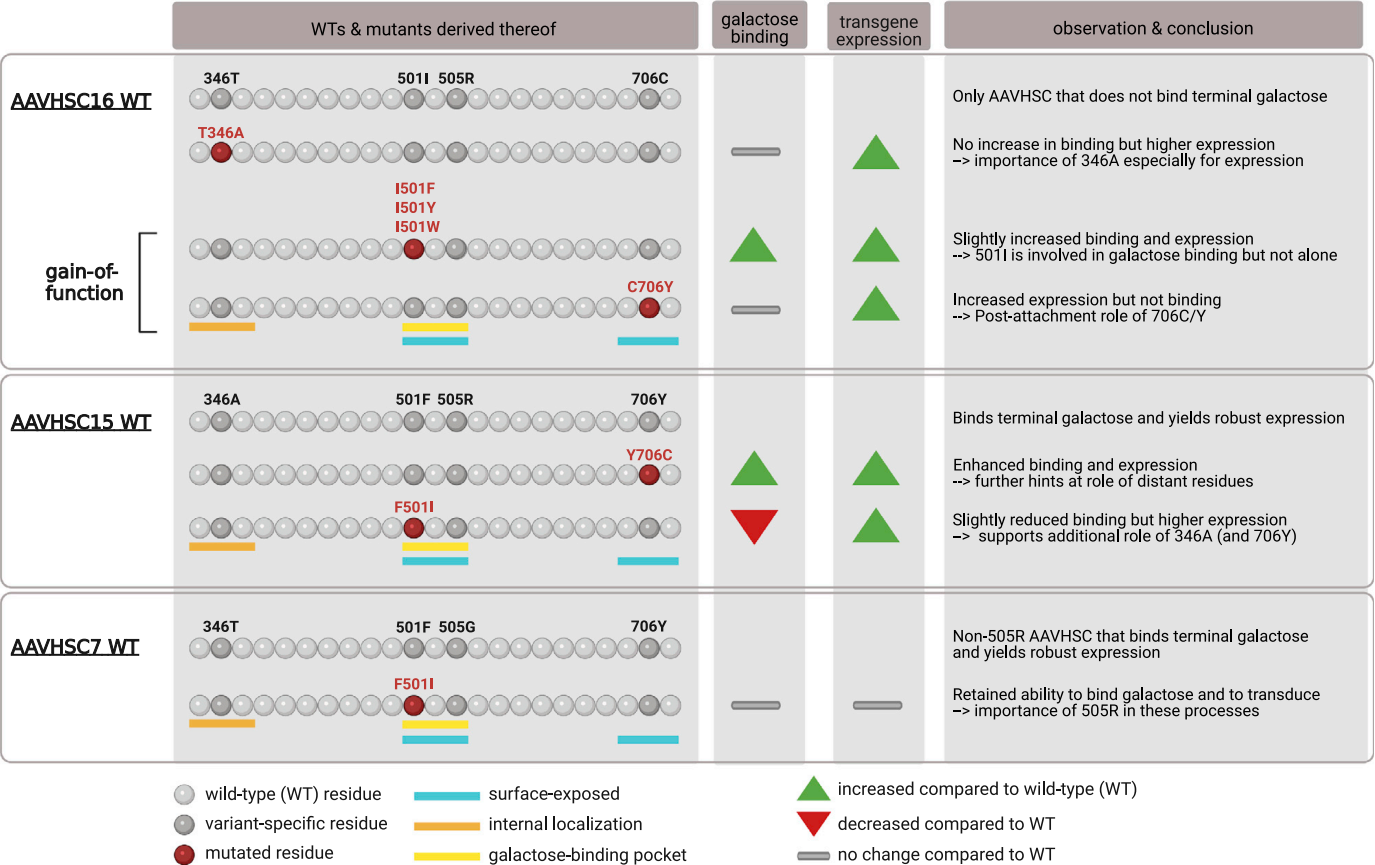
Schematic representation of wild-type or mutant AAVHSC capsid sequences and of their *in vitro* binding and expression properties as generated and studied by Smith and colleagues Note that neither are all residues shown nor are the colored bars indicating capsid regions drawn to scale. Instead, the bars highlight key residues within these (larger) regions. This figure was created with BioRender.com.

The current work by Smith et al. perfectly complements these and other prior studies in that it also harnesses comparison and mutagenesis of known AAV variants in order to identify singletons and inform the design of optimal AAV vectors. Therefore, the group focused on a subset of 11 AAVHSC variants, differing by up to four amino acids and known to exhibit distinct properties as vectors. Unexpectedly, new experiments revealed that AAVHSC16 shows no difference in binding to, or expression in, two CHO cell lines that differ in the extent of exposed galactose (Pro5: low; Lec2: high), whereas the other 10 AAVHSC variants preferably transduced the Lec2 cells. The fact that AAVHSC16 is distinguished from all other AAVHSCs and AAV9 by two unique residues, an isoleucine at position 501 (501I) and a cysteine at 706 (706C) (Figure 1 top), led the authors to dissect the contribution of these amino acids. Subsequent mutagenesis of AAVHSC16 as well as of AAVHSC15 and AAVHSC7 including their galactose-binding pockets allowed to define internal (346) and external (501, 505, and 706) capsid positions that are involved in binding, stabilization, intramolecular interactions, and transduction efficiency (Figure 1).

As noted, an isoleucine in position 501 is exclusively present in AAVHSC16, whereas AAV9 and other AAVHSCs carry an aromatic phenylalanine. Curiously, gain-of-function I501F mutation in AAVHSC16 only partially restored binding and expression in Lec2 cells compared with AAVHSC15, implying a critical role of the other positions 346 and 706 in which these two AAVHSC variants differ. Congruent with this, AAVHSC15 with the reverse mutation, F501I, expressed better than AAVHSC16 in all cell lines. Intriguingly, mutant AAVHSC16 C706Y did not rescue binding over the wild type but gave higher expression, implying a post-attachment mechanism. The reverse mutant AAVHSC15 Y706C bound and transduced Lec2 cells better than wild-type AAVHSC15, supporting the critical role of residues that are distantly located from the galactose-binding pocket and exemplifying the overall complexity of AAV biology. This was confirmed for position 346, whose T346A mutation in AAVHSC16 improved expression but not binding over the wild type.

Particularly interesting is, finally, position 505, as it allows us to further distinguish two AAVHSC subgroups that differ in binding to terminal galactose. One comprises AAVHSC13 and 15–17 that carry an arginine (505R) and bind galactose inefficiently, whereas an achiral glycine in AAVHSC1, 3–4, and 6–9 enhances galactose binding in Lec2 cells. The fact that mutant AAVHSC7 F501I still binds galactose and transduces Lec2 cells supports a pivotal role of 505G/R in these processes and strengthens the model that all four positions, 346, 501, 505, and 706, act together.

The most notable discovery is, however, that AAVHSC16 is liver detargeted in animals following peripheral administration of various doses, as consistently demonstrated in two mouse strains and in NHPs, using a variety of reporters and assays. Expression in peripheral tissues including the heart, muscle, and brain was comparable to the closely related AAVHSC15, illustrating the stunning propensity of single amino acids in the AAV capsid to mediate tissue specificity *in vivo*. This conclusion was verified in primary human hepatocytes, whose transduction with AAVHSC16 was improved by gain-of-function mutations at positions 501 and especially 706.

Last, but not least, the authors report the absence of detectable hepatotoxicity in AAVHSC16-treated NHPs. This is in stark contrast to animals injected with AAV9, AAVHSC8, or AAVHSC17, which all displayed elevated transaminase levels in their blood shortly after vector infusion.

Collectively, the results of this study support the notion of a complex, still incompletely understood sequence space of AAV capsids, where even alterations in a single residue (out of the ∼730 that make up the VP1 capsid protein) can readily produce surprising and strong effects. Combined with literature data, it is becoming increasingly clear that these variations can affect both cellular binding and transduction, but these properties are not necessarily linked. At the same time, this work compellingly illustrates how the rational, focused, and meticulous characterization of relevant residues can improve our understanding of AAV biology and thereby guide the development of better gene therapy vectors, combining efficiency with specificity and safety.

Until then, this work also leaves a series of open questions that need to be addressed as AAVHSC16 undergoes further preclinical evaluation. These include the fate of vectors that are not taken up by the liver and that may be cleared and shed through body fluids, as well as the immunoreactivity of AAVHSC variants with anti-AAV(9) neutralizing antibodies in humans. Most interesting will be to identify the glycan receptor(s) used by AAVHSC16, considering that it neither engages N-terminal galactose, mannose, acetyl-glucosamine, heparan sulfate proteoglycans, nor chondroitin sulfate. Present data combined with those in the literature imply that an aromatic ring side chain in position 501 is critical for interaction with a sugar moiety on target cells. This may not only aid in receptor identification, but it also once again exemplifies how basic and applied AAV research can synergize and ultimately benefit scientists, clinicians, and patients alike.
